# Orthodontia

**Published:** 1898-07

**Authors:** A. D. Williams


### THE DENTAL REGISTER.

VOL. LII.] JULY, 1898. [No. 7.

### COMMUNICATIONS.

### "Orthodontia."

BY A. D. WILLIAMS.

Read before Northwestern Ohio Dental Society, January 14, 1898.

The word "orthodontia" is derived from the Greek *orthos* straight, and *odovs* a tooth, and belongs exclusively to our vocabulary of dental literature; and is to-day recognized as that special branch of dental science which relates to the correction of abnormal positions of the human teeth.

That little or no attention has been given this subject until quite recently may be explained by the fact that the demands upon the dentist heretofore were principally, or probably exclusively, for the relief of pain, which consisted in the extraction of teeth and their replacement by artificial devices. But as the old system of astronomy satisfied the minds of philosophers in their day, so the old system of dentistry satisfied the practitioner in its day, but that day has passed, and we as professional and progressive dentists look forward to the time when the extraction of teeth (only in exceptional cases) will be a thing of the past.

No profession has in our time made more surprising strides than ours, and that which was once thought good practice is no longer considered as such. To-day, in the place of extracting and replacing, we try every known means to retain, and if decayed, renew the natural teeth. In a like manner we spare neither time or expense to endow the beautiful with that which she so much desires, namely, a beautiful denture; nature to her may probably not have been as kind as to others, but we would not criticise, rather assist her and by so doing add to our lists at least *one* grateful patient.

To know when a tooth is out of place in the arch we must know when it is in place. We therefore say, a tooth is in position when its diameter, meso-distally, is at right angles with a line drawn through the long axis of the tooth, and when its approximate surfaces are intact with its fellow at their most prominent points and its cusps properly occluding with those of the opposite jaw. Although any variation from this rule is an abnormality it does not follow that every tooth thus situated demands our attention; for instance, the overlapping of central incisors by laterals is in every sense an irregularity, but is so common that artifical teeth are often made to imitate it, and any effort at correction would seem folly. Here we may also mention misplaced molars, although out of position in the arch, occlusion is sufficient for all the requirements of mastication, and as appearance or speech are not interfered with there can be no good reason why *we* should interfere.

The etiology of these mal-positions may be classed under two general heads; namely, hereditary and acquired, while the former has probably for its greater cause inter-marriage. By this I mean one parent has a large frame, powerful jaw and teeth, while the other has the reverse. In the offspring we are apt to find the large jaw of the one and the small teeth of the other, or *vice versa*, which in one case means the over-crowding of the arch and in the other the abnormal inter-dental spaces. Under the head of acquired causes we have a long list which Dr. Guilford has classified as follows: Early extraction of deciduous teeth, too long retention of deciduous teeth, injudicious extraction of permanent teeth, delayed eruption of permanent teeth, supernumerary teeth, accidents, habits, adenoid growths.

As important as these acquired causes are, it would not be policy to here burden you with anything more than a few general remarks, especially in that which most practitioners consider the most important, namely, injudicious extraction. This is a very common mistake and cannot be too strongly criticised. We should never extract without the most careful consideration.

Since coming to Toledo the opportunities (at least to me) in this special branch have not been very encouraging, but with your permission for illustration I will give you one or two cases that came before my notice.

A very timid young lady, Miss B., aged fourteen, accompanied by her mother, presented herself at my office wishing the extraction of both upper cuspids which were erupting somewhat outside the arch. I tried to explain to both mother and patient the importance of retaining them, assuring both that nature in all probability would herself take care of them and bring them into place, all that was required was time. The young lady insisted on their extraction. I resisted, the consequence being that I lost both patient and money.

Another case somewhat similar came to the office of Dr. B. at this time. I was looking for a location and had been corresponding with this doctor, who had, as he said, one of the finest and best equipped offices in the State, and having more business than he could attend to wanted a partner. Thinking it might be a good thing I concluded to investigate and wrote him to that effect. I arrived at early Saturday morning and a couple of large gold teeth piloted me to the place. Scarcely had I introduced myself when a patient came in—boy about fifteen, both upper cuspids outside the arch. He, the doctor, looked at them and said, "They will have to come out." The next minute the old gentleman had the chloroform bottle and soon had his patient under its influence, when the teeth were extracted. About this time the boy's sister put in an appearance and cautioned the doctor against chloroform, which his physician had forbidden. But it was too late, it was all over, and those most important of all teeth were sacrificed through the ignorance of that old fogy who called himself a doctor. These people, in my estimation, do more to injure the profession (as well as the public) than we are aware. The expression of the individual depends so much upon the teeth that their loss is at least a very serious matter.

Man being omnivore, it is only reasonable to suppose that he has a combination of both the carnivore and herbivore, which he undoubtedly has; loss of the cuspid tooth is interference with the first of these, and absence of lateral incisors insures disappearance of the peaceful expression belonging to the second.

Among the various acquired causes of irregularities, habits are probably as common as any; those of thumb, lip and tongue-sucking cause a long string of deformities which would be almost impossible to enumerate. The evils associated with these malpositions are many, and vary from a slight marring of appearance to the incapacity of the teeth to perform their normal functions, namely, the mastication of food, which in time undermines and destroys the health of the individual.

Appearance, probably more than any one thing, has caused people to endure the operation necessary in the correction of teeth, and I can not do better than quote from Guilford the following paragraph. Referring to appearance, he says: "While this result is not probably the most important of those connected with irregularity, it is the one which most generally induces the patient to apply for remedial treatment. The other evils may not be recognized, or may be considered of minor importance to the patient, but the ill-appearance of the child both attracts the attention and enlists the sympathy to such an extent as to create a desire for its improvement."

Another important evil is the affection of speech. Every irregularity has its effect whither the arch is contracted and thus interferes with the movement of the tongue, or the teeth be separated so as to allow the air to escape, or any change in the alveolar ridge, it impairs speech. This demonstrates to us that it is as necessary for a person who wishes to become famous as a speaker or singer to have a good denture, as it is for the musician to have a good harp.

Irregular teeth have also a predisposition to caries, this is especially so when they are crowded. We have all probably had these decay and redecay, in spite of all we could do, until finally they were lost. The age at which correction should be attempted is also an important fact and should never be lost sight of. Success in all cases may not depend upon age, yet in earlier years it is much more easily accomplished, as the surrounding tissues have not reached that density which is attained at a later period.

This non-solidity of structure greatly favors the operation, but at the same time is poorly fitted to resist the predisposition of the teeth to again resume their former positions. Although between the thirteenth and eighteenth years is the time preferred by most practitioners, yet many who have given special attention to this branch of dentistry pay little attention to age, but as soon as a tooth shows misplacement which nature is not likely to correct, resort at once to mechanical means. After careful study it is the opinion of the writer that age should have due consideration with health and the extent of the operation, and while we would probably agree as to the most desirable age, we would not forget that this period, though the most favorable to the operator, is the most critical to the patient, for important changes are taking place and the vis-vitæ is taxed to the utmost. This is especially true of the female, so that we should never undertake at this period an operation which might injure the health of the individual. Better postpone. Health must never be sacrificed for any benefit conferred upon even the teeth. A knowledge of temperament is also important. For instance, a person of extreme nervous temperament is scarcely a proper subject for endurance of the details necessary for the correction of misplaced teeth.

After having considered the various points of advisability we take an impression, preferably of modeling compound, and fill it. This enables us to carefully consider our plan of procedure and definitely decide the method we shall use. As no method can be used in all cases the practitioner must be left to modify his treatment with every case, and is necessarily and wisely to be left to his own discretion and judgment.

The materials we have to select from are probably as variable as the different methods used, and are all the best thing in the right place and consist of the following: Piano-wire, platinum and its alloys, steel, German-silver, gold, vulcanite, compressed wood, ligatures and elastic rubber. We would not here attempt to discuss the virtues of these various materials, suffice to say that all appliances must be made of materials sufficient to do the work required, and in all cases be as simple as possible, easy to keep clean, inconspicuous and in no way injure the teeth. The last of these qualities is very important, and baffled for years the practitioner of this special branch, but when Dr. Magill gave to us the idea of cementing the appliances to the teeth he introduced a new era in regulating.

The many devices of Drs. Jackson, Byrnes, Angle, Magill, Coffin, Guilford, McCullum, Farrar, and others, are all good and very ingenious, and we as professional men appreciate their work and to them gladly give all honor. Orthodontia has come to stay, and in time can not help but attain that high position in our dental art which its importance demands.

In conclusion, I feel it my duty to credit the position in which we find the judicious practice of correcting irregularities a legitimate and most important part of dentistry, due greatly to the perseverance and untiring efforts of these gentlemen mentioned. By their methods and appliances we are enabled to perform successfully that branch of dental science so appropriately called "orthodontia."

